# Supplementary figures and images for: Circulating Exosomes Inhibit B Cell Proliferation and Activity
Source: Cancers (Basel). 2020 Jul 29;12(8):2110. doi: 10.3390/cancers12082110 (PMC7464446; doi:10.3390/cancers12082110)

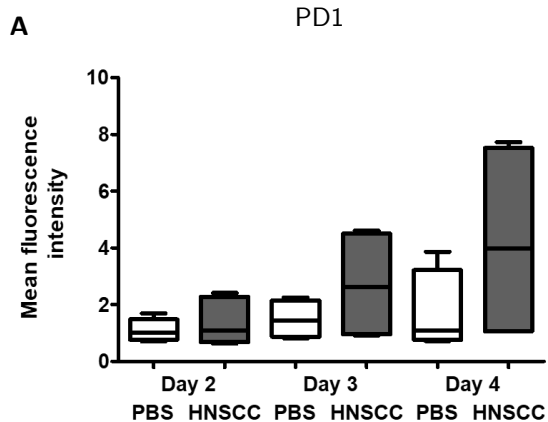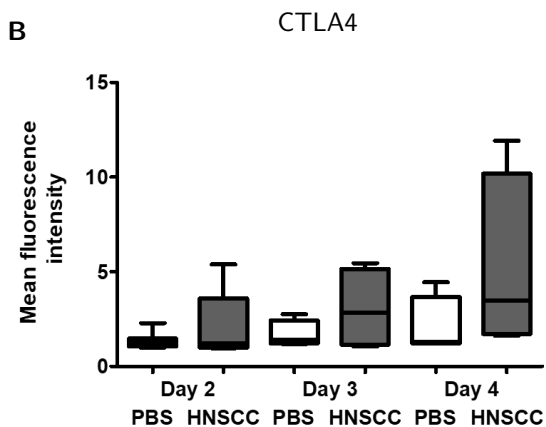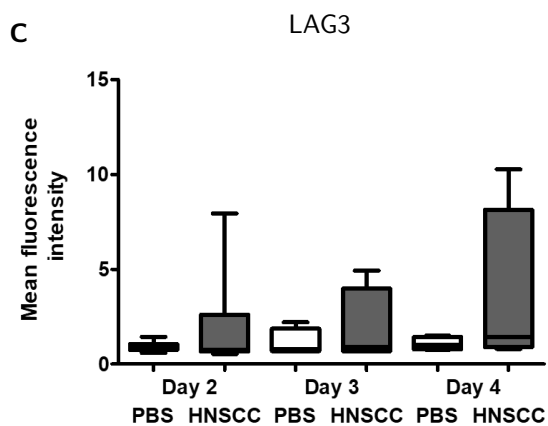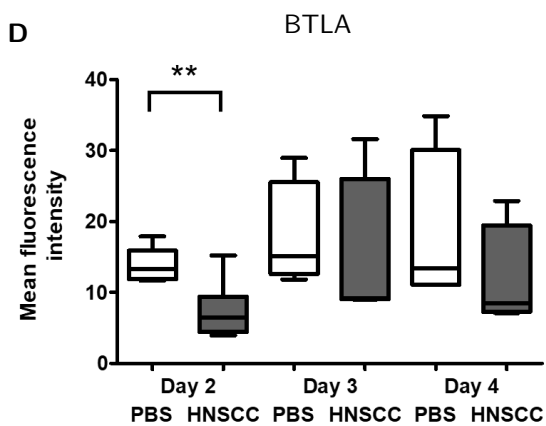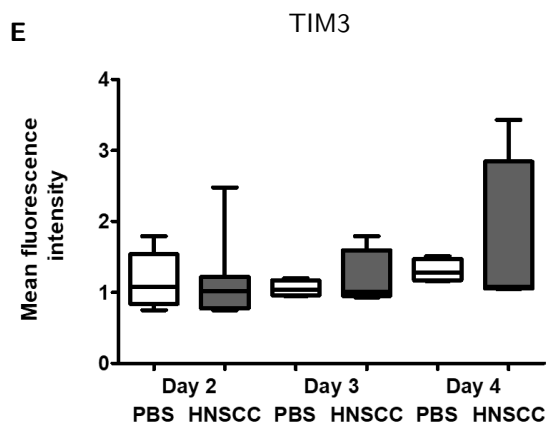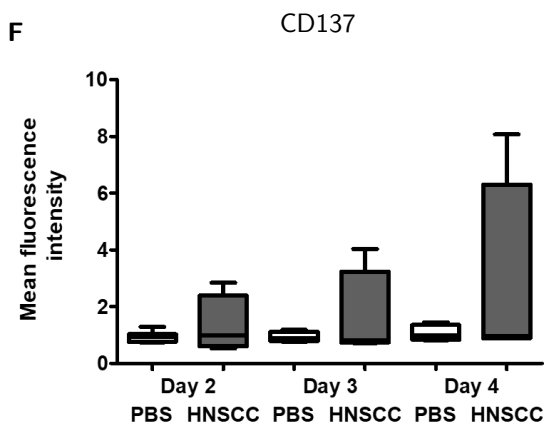

Supplement: Supplementary file 1 [file cancers-12-02110-s001.zip › Figure_S1.pdf]

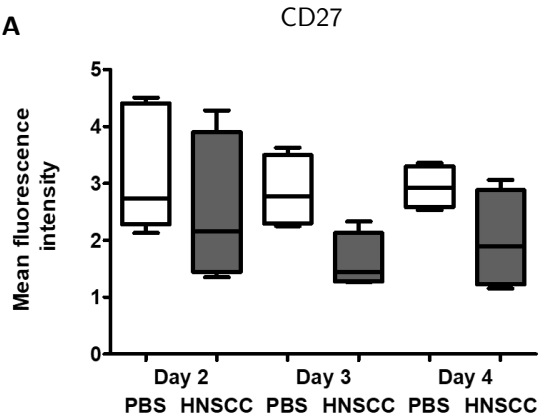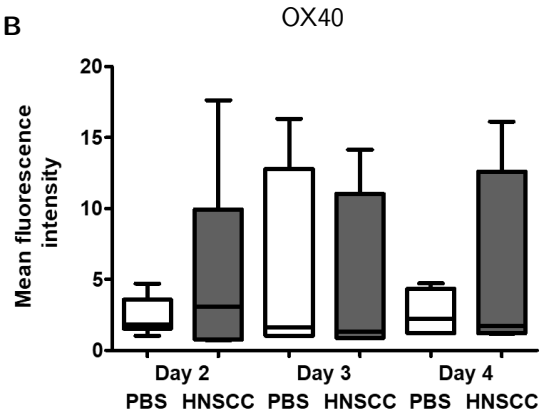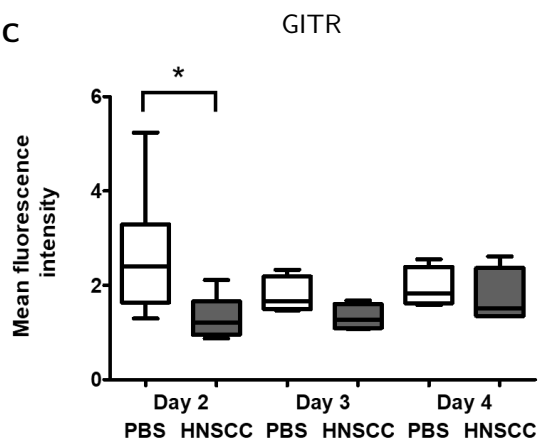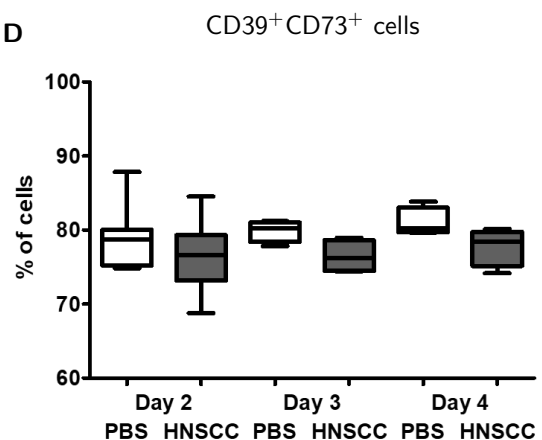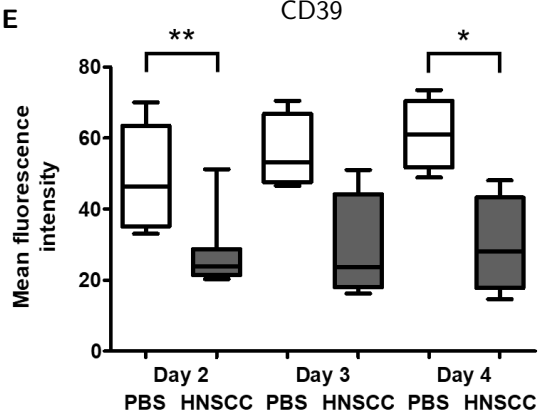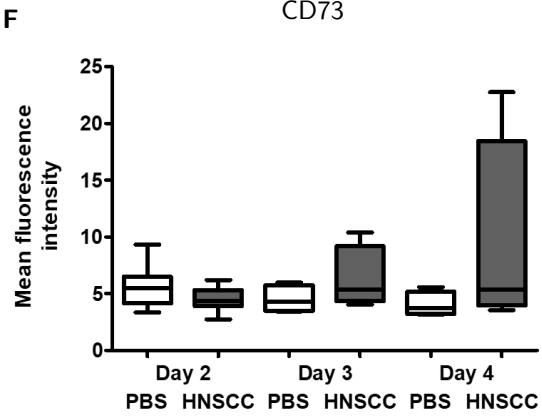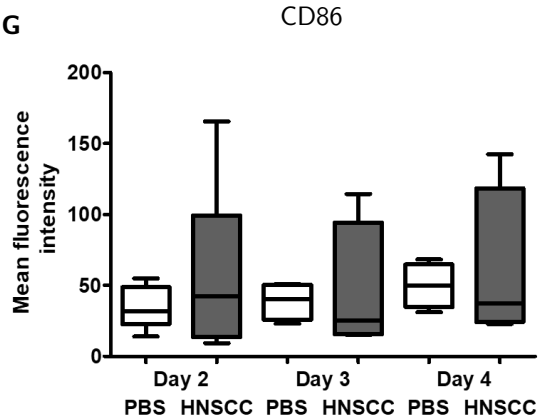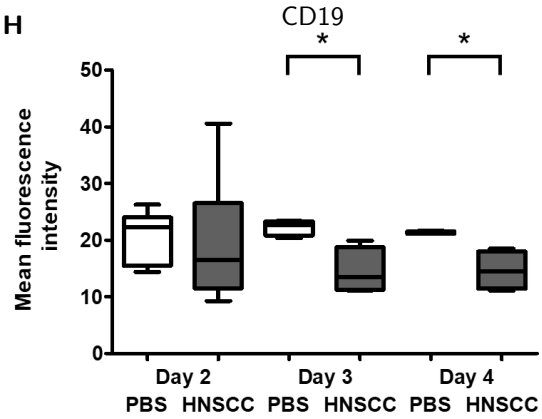

Supplement: Supplementary file 1 [file cancers-12-02110-s001.zip › Figure_S2.pdf]

A

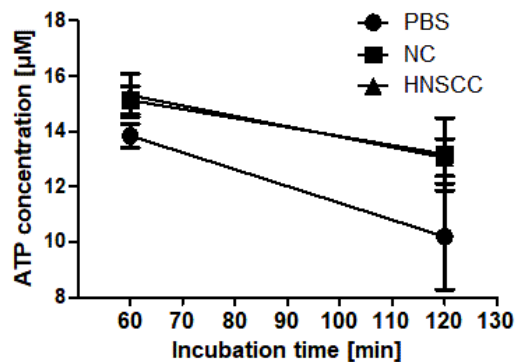

B

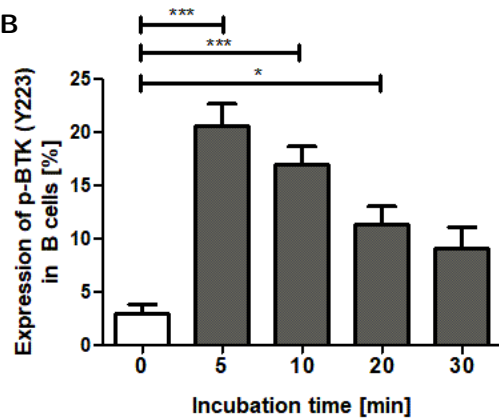

C

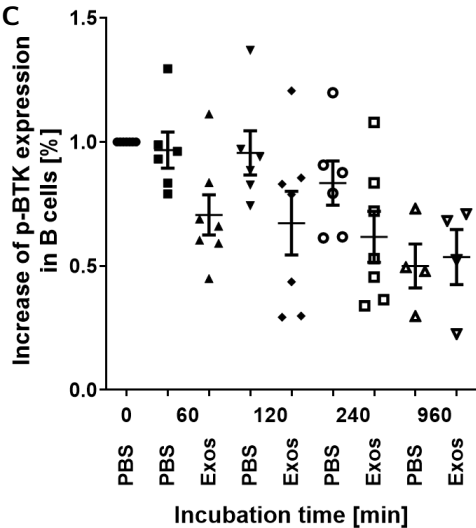

Increase of p-BTK expression  
in B cells [%]

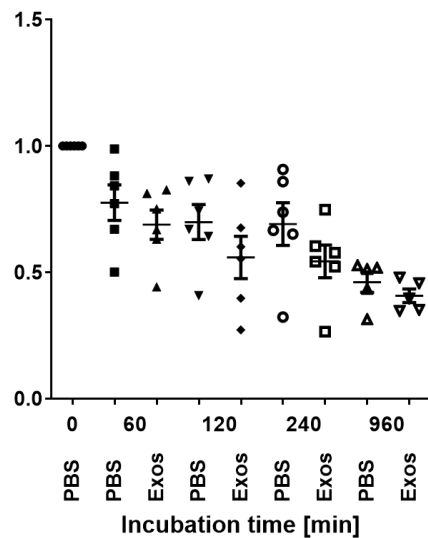

Supplement: Supplementary file 1 [file cancers-12-02110-s001.zip › Fig_S3.pdf]

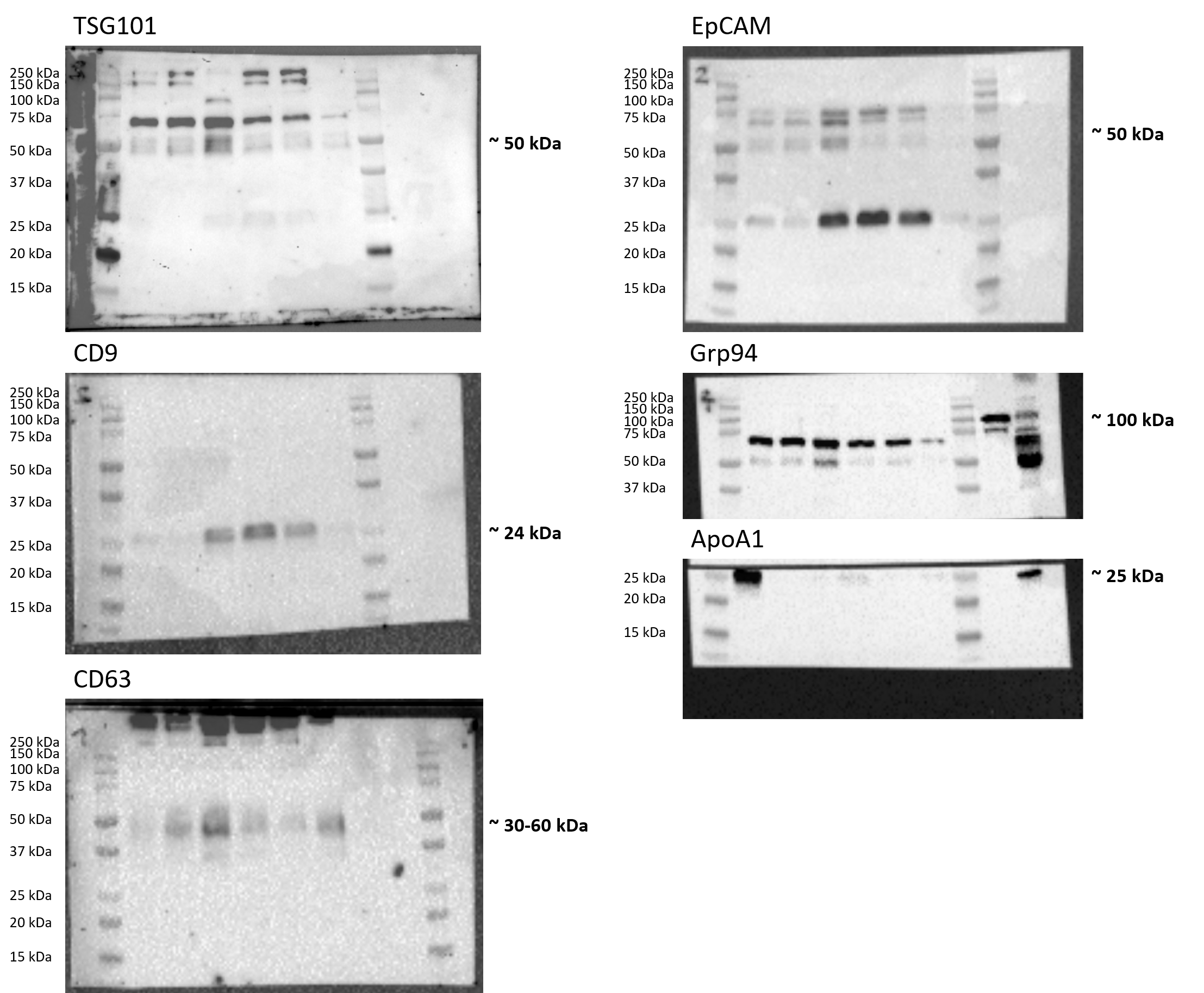

Supplement: Supplementary file 1 [file cancers-12-02110-s001.zip › Fig_S4.png]
